# Limited Prognostic Value of Pan-Immune-Inflammation Value Compared with NLR and Procalcitonin in Critically Ill Patients with Spontaneous Intracerebral Hemorrhage: A Retrospective Cohort Study

**DOI:** 10.3390/brainsci16080842

**Published:** 2026-08-08

**Authors:** İlkay Ceylan, Serpil Ekin, Nur Panik, Buket Özyaprak, Derful Gülen

**Affiliations:** Department of Anesthesiology and Reanimation, University of Health Sciences, Bursa Yüksek İhtisas Training and Research Hospital, 16310 Bursa, Türkiye; ceylanilkay81@gmail.com (İ.C.); drserpilekin@gmail.com (S.E.); nrgnlts@gmail.com (N.P.); ozyaprakertugrul@gmail.com (B.Ö.)

**Keywords:** intracerebral hemorrhage, pan-immune-inflammation value, PIV, neutrophil-to-lymphocyte ratio, NLR, inflammation, neurocritical care, mechanical ventilation, mortality, prognostic biomarkers

## Abstract

**Highlights:**

**What are the main findings?**

- Pan-immune-inflammation value showed limited prognostic performance and did not provide meaningful incremental prognostic value for mechanical ventilation requirement or 28-day mortality in critically ill patients with spontaneous intracerebral hemorrhage.
- Neutrophil-to-lymphocyte ratio and procalcitonin remained independently associated with adverse outcomes, with GCS score showing the highest observed discrimination for mechanical ventilation requirement and procalcitonin for 28-day mortality in this cohort.

**What are the implications of the main findings?**

- Among the evaluated inflammatory biomarkers, NLR and procalcitonin may be prioritized over PIV because they showed better prognostic performance in this cohort, with NLR additionally offering the advantages of low cost, universal availability, and straightforward calculation.
- Clinical severity assessment using GCS and the ICH score should remain the foundation of prognostic evaluation, while NLR and procalcitonin may provide complementary information for identifying patients who require closer monitoring and further assessment.

**Abstract:**

**Background:** Systemic inflammation plays a critical role in secondary brain injury after spontaneous intracerebral hemorrhage (ICH). The pan-immune-inflammation value (PIV) has recently emerged as a novel composite inflammatory biomarker; however, its prognostic significance in critically ill ICH patients remains unclear. **Methods:** This retrospective cohort study included 111 consecutive adult patients admitted to the intensive care unit (ICU) with spontaneous ICH between January 2020 and December 2024. PIV, neutrophil-to-lymphocyte ratio (NLR), platelet-to-lymphocyte ratio (PLR), and conventional clinical predictors were evaluated at ICU admission. The primary outcome was 28-day mortality, while the mechanical ventilation (MV) requirement was assessed as a secondary outcome. Receiver operating characteristic (ROC) analysis and logistic regression models were used to evaluate prognostic performance. **Results:** MV was required in 78 patients (70.3%), and 41 patients (36.9%) died within 28 days. Patients requiring MV had significantly higher PIVs than non-ventilated patients (*p* = 0.013), whereas PIVs did not differ significantly between survivors and non-survivors (*p* = 0.539). For predicting MV requirement, GCS score demonstrated the highest discriminative performance (AUC = 0.922), followed by ICH score (AUC = 0.874), procalcitonin (AUC = 0.861), and NLR (AUC = 0.832), whereas PIV showed modest discrimination (AUC = 0.650). For 28-day mortality, procalcitonin achieved the highest AUC (0.800), while PIV showed poor predictive performance (AUC = 0.535). In multivariable analysis, NLR and procalcitonin remained independently associated with both MV requirement and 28-day mortality. PIV was not included in the final multivariable models because of its overlap with NLR; moreover, it did not provide meaningful incremental prognostic value when added to the reference models. **Conclusions:** In this single-center cohort of ICU-admitted patients with spontaneous ICH, PIV did not add meaningful prognostic value beyond established clinical and inflammatory predictors. These findings do not support the routine use of PIV for early risk stratification in this specific population; simpler markers, particularly NLR and procalcitonin, may be preferred when interpreted alongside established clinical severity measures.

## 1. Introduction

Intracerebral hemorrhage (ICH) is one of the most devastating subtypes of stroke, accounting for 10–15% of all stroke cases while contributing disproportionately to stroke-related mortality and long-term disability [1]. Spontaneous ICH refers to non-traumatic bleeding into the brain parenchyma [1]. The present study was restricted to spontaneous ICH to maintain etiological and clinical homogeneity and to avoid combining patients with traumatic hemorrhage, who represent a different clinical population. Globally, ICH results in over three million deaths annually and remains among the leading causes of death and severe neurological disability, with a 30-day case fatality rate approaching 40% [1]. Despite advances in neurocritical care and neurosurgical management, outcomes have improved only modestly over recent decades, and early identification of patients at high risk for clinical deterioration remains essential for timely clinical decision-making [1].

Several clinical and radiological variables—including age, Glasgow Coma Scale (GCS) score, hematoma volume, intraventricular hemorrhage (IVH), and the ICH score—have been established as major determinants of outcome following spontaneous ICH [2]. However, growing evidence indicates that secondary brain injury mediated by complex neuroinflammatory mechanisms plays a critical and independent role in neurological deterioration beyond the initial structural insult [3,4].

Following the bleeding event, microglial activation, infiltration of circulating leukocytes—particularly neutrophils—release of pro-inflammatory cytokines, including interleukin-1β and tumor necrosis factor-α, and blood–brain barrier disruption collectively promote perihematomal edema formation and progressive neuronal injury [3,5]. These systemic immunological perturbations are readily detectable in the peripheral blood, providing the biological rationale for the clinical use of hematological inflammation-based indices as prognostic biomarkers.

Among blood-derived inflammatory indices, the neutrophil-to-lymphocyte ratio (NLR) has been the most extensively studied in ICH. Multiple cohort studies and systematic reviews have demonstrated consistent associations between elevated NLR and mortality, poor neurological outcomes, hematoma expansion, and secondary complications [6,7]. More recent evidence from a large meta-analysis was also consistent with the prognostic utility of NLR for both short-term mortality and neurological dysfunction following spontaneous ICH [8].

The pan-immune-inflammation value (PIV), calculated as (neutrophil count × monocyte count × platelet count)/lymphocyte count, is an empirically derived composite index incorporating four peripheral blood-cell populations [9]. Although this broader cellular composition may provide a multidimensional representation of systemic immune activation, the inclusion of additional components does not necessarily imply greater biological specificity or better predictive performance. Each component of PIV may be influenced by acute critical illness–related factors, including infection, physiological stress, tissue injury, surgical interventions, medication exposure, hemodilution, and platelet consumption. Accordingly, PIV should be regarded as a pragmatic blood count–derived marker rather than a direct mechanistic measure of neuroinflammation.

PIV was initially introduced in oncology [9] and has subsequently been evaluated in cardiovascular disease [10] and spontaneous ICH [11], including as part of a proposed short-term prognostic nomogram. However, evidence regarding the prognostic performance of PIV in patients admitted to the intensive care unit (ICU) with spontaneous ICH remains limited, and it is unclear whether PIV provides clinically meaningful information beyond simpler markers such as NLR and established clinical severity measures. Against this background, the present study differs from previous reports in several respects. First, it specifically focuses on critically ill patients with spontaneous ICH requiring ICU admission. Second, rather than evaluating PIV in isolation or as part of a prognostic nomogram, it directly compares PIV with established inflammatory indices, including NLR and the platelet-to-lymphocyte ratio (PLR), as well as procalcitonin and conventional clinical and radiological predictors. Third, it evaluates two clinically distinct outcomes—mechanical ventilation (MV) requirement and 28-day mortality—to determine whether PIV provides incremental prognostic information in a high-risk neurocritical care population.

Therefore, this study aimed to: (i) evaluate the predictive performance of PIV measured at ICU admission for MV requirement and 28-day mortality in critically ill patients with spontaneous ICH; (ii) compare the discriminative accuracy of PIV with NLR, PLR, and conventional clinical predictors using ROC curve analysis; and (iii) evaluate adjusted associations between clinically selected predictors and the study outcomes using multivariable logistic regression.

## 2. Materials and Methods

### 2.1. Study Design and Population

This retrospective observational cohort study was conducted in the intensive care unit of a tertiary-care university hospital. Adult patients (≥18 years) admitted to the ICU with spontaneous intracerebral hemorrhage between January 2020 and December 2024 were screened for eligibility. For the purposes of this study, spontaneous ICH was defined as an acute non-traumatic intraparenchymal hemorrhage confirmed by computed tomography at admission. Patients were excluded if they had hemorrhage secondary to trauma, vascular malformation, aneurysm, intracranial tumor, or hemorrhagic transformation of ischemic stroke; subarachnoid hemorrhage; active malignancy or hematological disorders; incomplete laboratory data precluding calculation of inflammatory indices; or missing primary outcome data.

The study protocol was submitted to the Medical Sciences Ethics Committee of Bursa Yüksek İhtisas Training and Research Hospital, University of Health Sciences, on 23 March 2026 and approved on 8 April 2026 (approval no. 2026/04-02; protocol code: 2024-TBEK). Patient records were accessed for research purposes and analyzed only after ethics committee approval had been obtained. The study was conducted in accordance with the Declaration of Helsinki.

### 2.2. Data Collection

Demographic characteristics, clinical findings, laboratory parameters, radiological data, and outcome variables were retrospectively collected from electronic medical records and the hospital information system. The following demographic, clinical, and radiological variables were recorded at ICU admission: age, sex, GCS score, systolic blood pressure (SBP), comorbidities (hypertension and diabetes mellitus), medication history (anticoagulant and antiplatelet use), hematoma volume calculated using the ABC/2 method, presence of IVH, and infratentorial localization. ICH-related neurosurgical intervention performed during the index hospitalization was recorded as a binary variable (yes/no). This variable did not include previous surgical history or procedures involving other organ systems. Because the exact temporal sequence between neurosurgical intervention and initiation of mechanical ventilation could not be consistently determined from the retrospective records, surgery was defined as an intervention occurring at any time during the index hospitalization and was not interpreted as necessarily preceding mechanical ventilation. The ICH score was calculated according to the original Hemphill criteria [2].

Laboratory parameters included complete blood count variables—white blood cell (WBC), neutrophil, lymphocyte, monocyte, and platelet counts—as well as C-reactive protein (CRP), procalcitonin, and lactate, all measured at ICU admission. The exact interval between symptom onset and blood sampling, as well as the temporal relationship of sampling to endotracheal intubation and surgical or other invasive procedures, could not be consistently determined from the retrospective medical records. Inflammatory indices were calculated as follows:

***NLR = Neutrophil count/Lymphocyte count***

***PLR = Platelet count/Lymphocyte count***

***PIV = (Neutrophil × Monocyte × Platelet)/Lymphocyte***

### 2.3. Outcomes

The primary outcome was 28-day all-cause mortality. The secondary outcome was the requirement for invasive mechanical ventilation during the ICU stay. For the purposes of this study, invasive mechanical ventilation was defined as endotracheal intubation followed by invasive positive-pressure ventilation at any time during the ICU stay. This definition was intended to encompass ventilation initiated at ICU admission as well as ventilation initiated later during the ICU course. Because the exact time from ICU admission to intubation, the standardized indications for intubation, and the duration of mechanical ventilation could not be consistently determined from the retrospective records, mechanical ventilation was analyzed as a binary outcome—required versus not required—rather than as a time-to-event or duration-based variable.

### 2.4. Statistical Analysis

Statistical analyses were performed using Python version 3.11 (Python Software Foundation, Wilmington, DE, USA), SciPy version 1.11, and scikit-learn version 1.3. The Shapiro–Wilk test was used to assess normality of continuous variables. Normally distributed variables are presented as mean ± standard deviation (SD); non-normally distributed continuous variables as median with interquartile range (IQR: 25th–75th percentile); and categorical variables as counts with percentages. No statistical imputation was performed for missing data. Patients with missing laboratory values required for calculation of PIV, NLR, or PLR or with missing 28-day mortality data were excluded. All analyses were therefore conducted using complete cases.

Between-group comparisons of continuous variables were performed using the Mann–Whitney U test. Categorical variables were compared using the Pearson chi-square test with Yates’ continuity correction or Fisher’s exact test, as appropriate. Spearman rank correlation analysis was conducted to assess associations between PIV and other inflammatory indices. A two-sided *p*-value < 0.05 was considered statistically significant.

Receiver operating characteristic (ROC) curve analysis was performed to evaluate the discriminative accuracy of PIV, NLR, PLR, GCS score, ICH score, procalcitonin, and hematoma volume for predicting MV requirement and 28-day mortality. For GCS score, lower values were treated as indicating higher risk so that AUC values reflected discrimination in the clinically expected direction. Area under the curve (AUC) values with 95% confidence intervals (CIs) were estimated by bootstrap resampling (2000 iterations, percentile method). Optimal cutoff values were determined by the Youden index (sensitivity + specificity − 1). Prespecified pairwise comparisons between PIV and selected established markers—NLR, GCS score, and ICH score—were performed using two-sided bootstrap-based testing with 2000 resamples, and the results are presented in Supplementary Table S1.

To evaluate the incremental prognostic value of PIV, nested logistic regression models were constructed separately for mechanical ventilation requirement and 28-day mortality. A reference model including the standard ICH score, NLR, and procalcitonin was compared with an extended model additionally incorporating PIV. Because of their right-skewed distributions, NLR, procalcitonin, and PIV were transformed using ln(x + 1). Incremental model performance was assessed by changes in the c-statistic, bootstrap-based comparison of AUCs using 2000 resamples, likelihood-ratio testing, Brier scores, and Hosmer–Lemeshow goodness-of-fit testing.

Univariable logistic regression analyses were performed to describe the individual associations of candidate predictors with mechanical ventilation requirement and 28-day mortality. Predictor selection for the final multivariable models was not based on univariable statistical significance. Instead, both final models used the same clinically driven fixed predictor set comprising GCS score, ICH score, hematoma volume, NLR, and procalcitonin, representing neurological severity, radiological burden, and systemic inflammatory response. The final models included the main effects of these predictors only. No interaction terms were evaluated because no specific interaction hypothesis had been prespecified and the limited number of outcome events would have increased the risk of overfitting and unstable estimates. PLR was not included in the prespecified multivariable models because NLR was selected as the primary inflammation-based marker based on prior evidence and clinical interpretability. ICH-related neurosurgical intervention was not included in the final multivariable models because it represented a treatment exposure occurring during the index hospitalization rather than a baseline prognostic variable. Its temporal relationship with mechanical ventilation could not be consistently established, and selection for surgery was likely influenced by hematoma characteristics and clinical severity. Additional variables were not entered solely on the basis of statistical significance in the univariable analyses. Multicollinearity was assessed using variance inflation factors (VIFs) and tolerance values. A VIF < 5 and a tolerance value > 0.20 were considered indicative of acceptable collinearity. Because PIV and NLR demonstrated substantial mathematical and statistical overlap, they were not entered simultaneously into the final multivariable coefficient models. PIV was instead evaluated for adjusted incremental prognostic value through the nested reference and extended models described above. For logistic regression analyses, right-skewed inflammatory biomarkers were transformed using ln(x + 1), after which continuous predictors were standardized to z-scores. Accordingly, odds ratios (ORs) and adjusted odds ratios (aORs) for continuous variables represent the change in outcome odds associated with a one-standard-deviation increase in the corresponding predictor; for log-transformed biomarkers, this refers to a one-standard-deviation increase in the ln(x + 1)-transformed value. Binary variables were coded as present versus absent. Two-sided *p*-values for logistic regression coefficients were obtained using Wald tests, whereas confidence intervals for the multivariable models were estimated using bootstrap resampling with 2000 iterations. During each bootstrap iteration, the prespecified final predictor set was refitted in the resampled dataset; variable selection was not repeated within individual bootstrap samples. Accordingly, this procedure assessed the stability of the prespecified fitted models but did not incorporate uncertainty arising from the broader model-specification process. Model discrimination was quantified using the c-statistic.

## 3. Results

### 3.1. Patient Characteristics

A total of 111 patients with spontaneous intracerebral hemorrhage were included in the final analysis. The median age was 69.0 years (IQR: 60.5–79.0), and 68 patients (61.3%) were male. The median Glasgow Coma Scale (GCS) score was 8 (IQR: 4–13), the median ICH score was 2 (IQR: 2–4), and the median hematoma volume was 84.0 mL (IQR: 40.2–171.8). Mechanical ventilation was required in 78 patients (70.3%), and 41 patients (36.9%) died within 28 days. All 41 deaths occurred among patients who required mechanical ventilation, corresponding to a 28-day mortality rate of 52.6% (41/78) in the mechanically ventilated group, whereas no deaths occurred among the 33 patients who did not require mechanical ventilation. Median admission PIV, NLR, and PLR values were 901.6 (457.7–2019.9), 11.3 (6.5–17.2), and 216.8 (159.9–323.3), respectively. Baseline characteristics according to mechanical ventilation requirement and 28-day mortality are presented in Table 1 and Table 2, respectively.

Patients requiring mechanical ventilation were significantly older and exhibited lower GCS scores, higher ICH scores, larger hematoma volumes, higher neutrophil counts, lower lymphocyte counts, and higher inflammatory marker levels compared with those who did not require ventilatory support. Significant differences were observed for PIV (*p* = 0.013), NLR (*p* < 0.001), PLR (*p* = 0.002), CRP (*p* < 0.001), and procalcitonin (*p* < 0.001).

Non-survivors were significantly older and had lower GCS scores, higher ICH scores, larger hematoma volumes, lower lymphocyte counts, higher NLR values, and elevated procalcitonin levels compared with survivors. In contrast, PIVs were comparable between mortality groups and did not demonstrate a significant association with 28-day mortality (*p* = 0.539).

### 3.2. ROC Curve Analysis

ROC analysis demonstrated excellent predictive performance of GCS score for mechanical ventilation requirement (AUC = 0.922, 95% CI 0.858–0.974), followed by ICH score (AUC = 0.874), procalcitonin (AUC = 0.861), NLR (AUC = 0.832), and hematoma volume (AUC = 0.797). PIV showed only modest discrimination (AUC = 0.650).

For 28-day mortality, procalcitonin demonstrated the highest predictive accuracy (AUC = 0.800), followed by hematoma volume (AUC = 0.691), NLR (AUC = 0.687), and ICH score (AUC = 0.672). PIV exhibited poor discriminatory performance with an AUC of 0.535.

**Figure 1:**
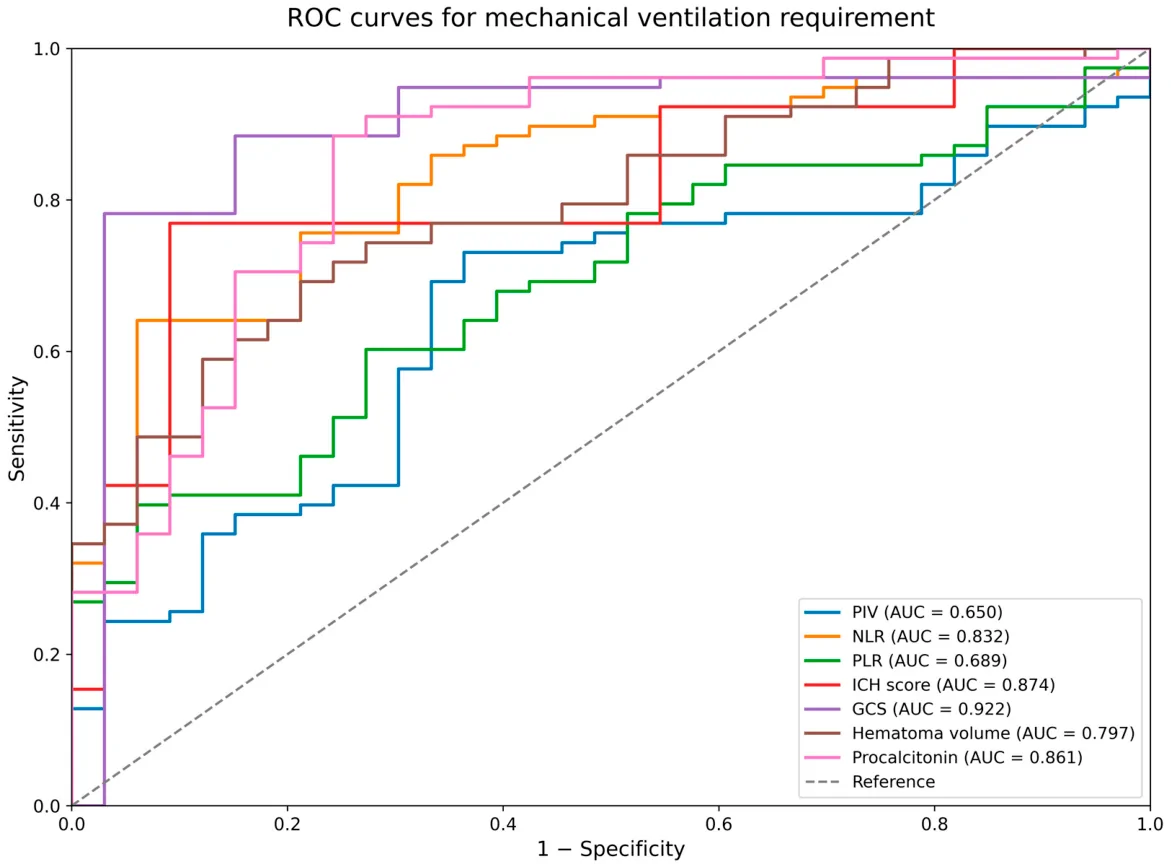
Receiver operating characteristic (ROC) curves for prediction of mechanical ventilation requirement. ROC curves comparing the discriminative performance of PIV, NLR, PLR, GCS score, ICH score, procalcitonin, and hematoma volume for predicting mechanical ventilation requirement. GCS score demonstrated the highest discriminative accuracy (AUC = 0.922), followed by ICH score (AUC = 0.874), procalcitonin (AUC = 0.861), and NLR (AUC = 0.832), whereas PIV showed only modest discrimination (AUC = 0.650).

**Figure 2:**
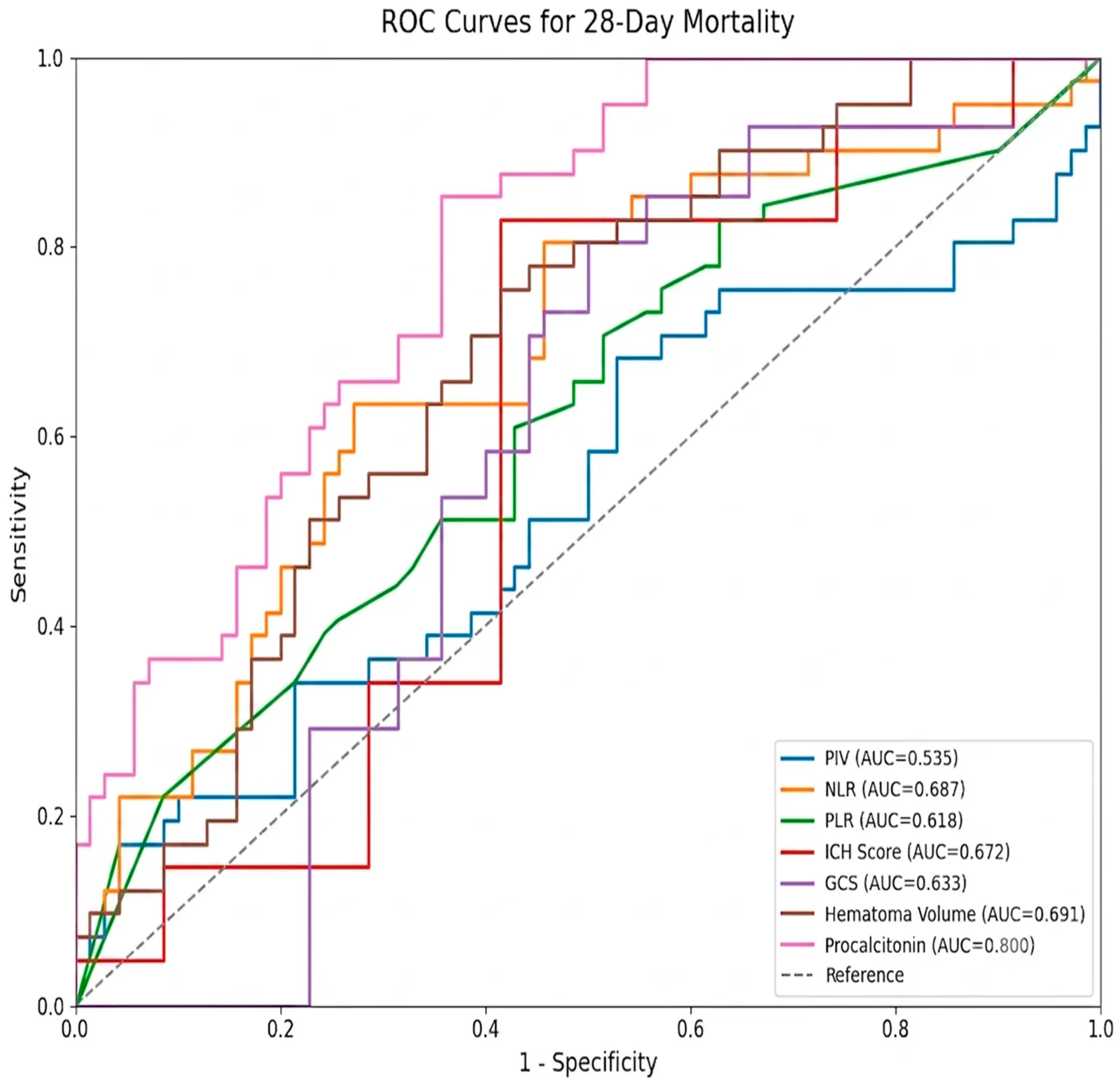
Receiver operating characteristic (ROC) curves for prediction of 28-day mortality. ROC curves comparing the discriminative performance of inflammatory biomarkers and clinical variables for predicting 28-day mortality. Procalcitonin demonstrated the highest predictive accuracy (AUC = 0.800), followed by hematoma volume (AUC = 0.691), NLR (AUC = 0.687), and ICH score (AUC = 0.672). PIV showed poor discriminatory performance (AUC = 0.535).

Pairwise ROC curve comparisons were performed using two-sided bootstrap-based testing with 2000 resamples. For mechanical ventilation requirement, the AUCs of NLR (AUC difference = 0.183, *p* = 0.0002), GCS score (AUC difference = 0.266, *p* < 0.0001), and ICH score (AUC difference = 0.220, *p* = 0.0006) were significantly higher than that of PIV. For 28-day mortality, NLR also showed a significantly higher AUC than PIV (AUC difference = 0.152, *p* = 0.0016), whereas the differences between PIV and GCS score (AUC difference = 0.103, *p* = 0.167) or ICH score (AUC difference = 0.141, *p* = 0.082) were not statistically significant. The corresponding ROC curves are shown in Figure 1 and Figure 2, and detailed performance metrics are presented in Table 3 and Table 4. Complete results are presented in Supplementary Table S1.

### 3.3. Incremental Prognostic Value of PIV

Nested model analyses were performed to determine whether PIV provided additional prognostic information beyond established clinical and inflammatory predictors. For mechanical ventilation, addition of PIV to the reference model did not improve discrimination (AUC 0.916 vs. 0.916; ΔAUC = −0.0004, bootstrap *p* = 0.463), although a nominal improvement in model fit was observed (likelihood-ratio test, *p* = 0.044).

For 28-day mortality, inclusion of PIV resulted in a small numerical increase in discrimination, with the AUC increasing from 0.789 to 0.811 (ΔAUC = 0.023); however, this improvement was not statistically significant (bootstrap *p* = 0.268; likelihood-ratio test, *p* = 0.052). Calibration metrics were similar between the reference and extended models. For mechanical ventilation, the reference and extended models had Brier scores of 0.107 and 0.106, respectively, and the Hosmer–Lemeshow test did not indicate evidence of poor fit (*p* = 0.168 and *p* = 0.808, respectively). For 28-day mortality, the corresponding Brier scores were 0.173 and 0.167, with Hosmer–Lemeshow *p*-values of 0.654 and 0.529, respectively. These results did not indicate substantial calibration concerns, although the calibration estimates should be interpreted cautiously given the limited sample size. Overall, PIV did not provide meaningful incremental prognostic value beyond the established predictors included in the reference models (Supplementary Table S2).

### 3.4. Univariable Logistic Regression Analysis

In the order presented in Table 5, lower GCS score (OR = 0.06, 95% CI 0.02–0.19, *p* < 0.001), larger hematoma volume (OR = 7.71, 95% CI 2.71–21.96, *p* < 0.001), higher ICH score (OR = 5.94, 95% CI 2.86–12.36, *p* < 0.001), and ICH-related neurosurgical intervention (OR = 64.00, 95% CI 8.28–494.82, *p* < 0.001) were significantly associated with mechanical ventilation requirement in univariable analyses. Higher NLR (OR = 4.64, 95% CI 2.42–8.89, *p* < 0.001), PLR (OR = 1.96, 95% CI 1.19–3.24, *p* = 0.009), procalcitonin (OR = 19.43, 95% CI 2.55–147.92, *p* = 0.004), neutrophil count (OR = 2.04, 95% CI 1.16–3.57, *p* = 0.013), and CRP (OR = 2.56, 95% CI 1.57–4.20, *p* < 0.001) were also significantly associated with mechanical ventilation requirement, whereas higher lymphocyte count was associated with lower odds of this outcome (OR = 0.47, 95% CI 0.24–0.93, *p* = 0.030). Age and IVH were not statistically significant in the univariable analysis. PIV was explicitly evaluated but was not significantly associated with mechanical ventilation requirement (OR = 1.49, 95% CI 0.96–2.32, *p* = 0.074).

In the order presented in Table 6, older age (OR = 1.77, 95% CI 1.14–2.77, *p* = 0.012), lower GCS score (OR = 0.60, 95% CI 0.40–0.91, *p* = 0.015), larger hematoma volume (OR = 1.83, 95% CI 1.18–2.82, *p* = 0.006), higher ICH score (OR = 1.95, 95% CI 1.26–3.01, *p* = 0.003), and ICH-related neurosurgical intervention (OR = 3.26, 95% CI 1.46–7.32, *p* = 0.004) were significantly associated with 28-day mortality. Higher NLR (OR = 2.09, 95% CI 1.29–3.38, *p* = 0.003), PLR (OR = 1.61, 95% CI 1.05–2.47, *p* = 0.028), and procalcitonin (OR = 3.39, 95% CI 1.73–6.65, *p* < 0.001) were also significantly associated with mortality, whereas higher lymphocyte count was associated with lower mortality odds (OR = 0.26, 95% CI 0.11–0.63, *p* = 0.003). IVH, neutrophil count, and CRP were not statistically significant. PIV was explicitly evaluated but was not significantly associated with 28-day mortality (OR = 1.05, 95% CI 0.71–1.55, *p* = 0.807).

### 3.5. Multivariable Logistic Regression Analysis

Collinearity diagnostics were performed before fitting the final multivariable models. VIF values were 2.73 for GCS score, 2.93 for ICH score, 1.28 for hematoma volume, 1.01 for NLR, and 1.06 for procalcitonin, with corresponding tolerance values ranging from 0.341 to 0.986. These findings indicated no problematic multicollinearity among the variables retained in the final models. In contrast, when PIV and NLR were considered simultaneously, their VIF values increased to 6.28 and 6.29, respectively, supporting the decision not to include both indices in the same final model. Detailed collinearity diagnostics are presented in Supplementary Table S3. Accordingly, PIV was not omitted from prognostic evaluation; rather, its incremental contribution was assessed using the nested models reported in Section 3.3. Its individual adjusted coefficient was not interpreted in the final coefficient models because simultaneous inclusion with NLR resulted in elevated VIF values and potentially unstable coefficient estimates.

After adjustment for GCS score, ICH score, hematoma volume, NLR, and procalcitonin, lower GCS score (adjusted OR [aOR] 0.200, 95% CI 0.087–0.415, *p* < 0.001), larger hematoma volume (aOR 2.200, 95% CI 1.202–4.443, *p* = 0.018), elevated NLR (aOR 2.430, 95% CI 1.288–4.870, *p* = 0.009), and higher procalcitonin levels (aOR 1.984, 95% CI 1.298–3.132, *p* = 0.002) remained independently associated with mechanical ventilation requirement. The model demonstrated excellent discrimination (c-statistic = 0.944).

For 28-day mortality, NLR (aOR 3.192, 95% CI 1.271–7.652, *p* = 0.011) and procalcitonin (aOR 5.297, 95% CI 2.184–10.100, *p* < 0.001) remained independently associated with mortality, whereas GCS score, ICH score, and hematoma volume lost statistical significance. The mortality model demonstrated good discrimination (c-statistic = 0.809). Detailed adjusted estimates are presented in Table 7.

**Figure 3:**
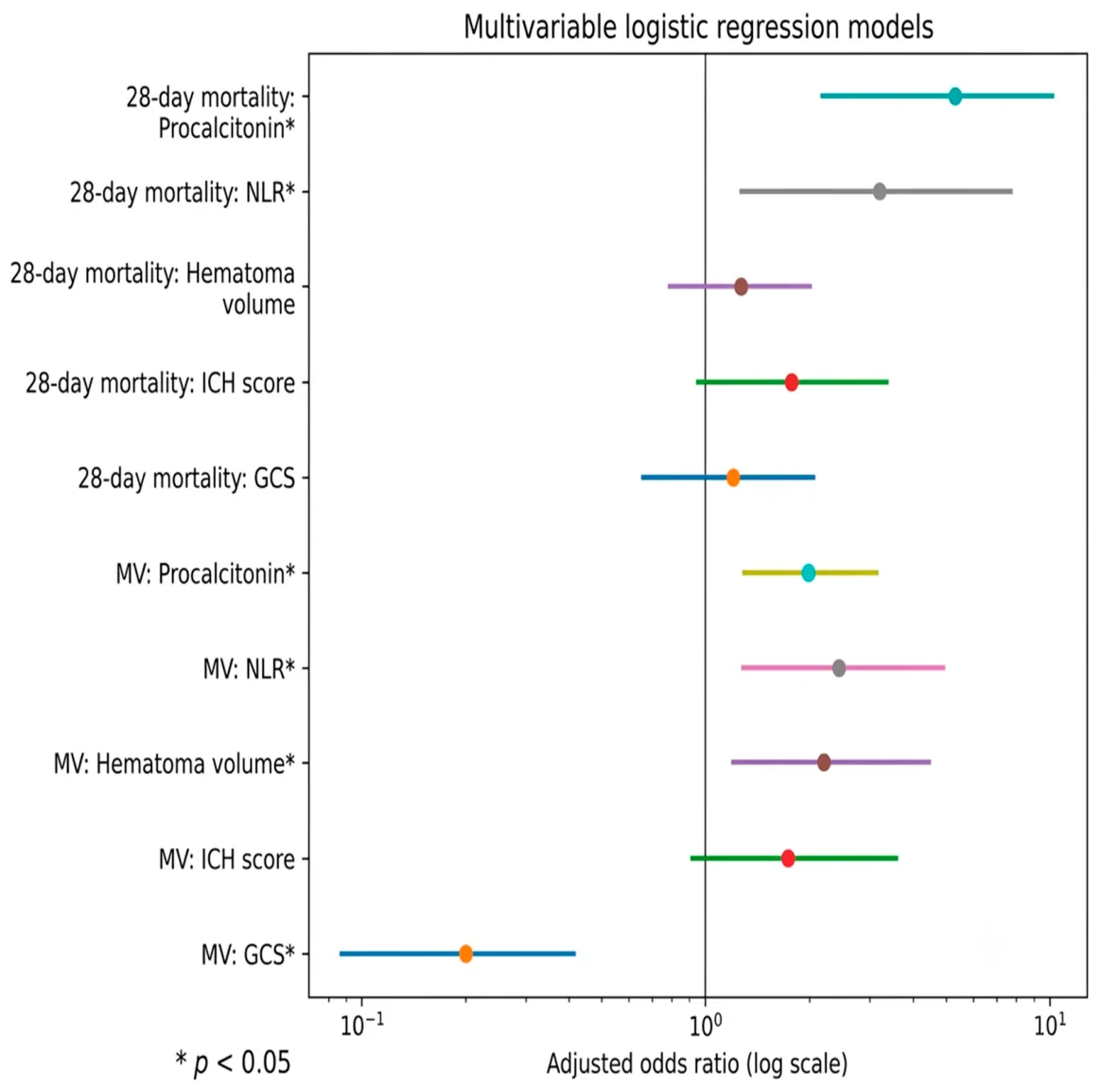
Forest plot of multivariable logistic regression models for mechanical ventilation requirement and 28-day mortality. Forest plot showing adjusted odds ratios (aORs) and 95% confidence intervals for all variables included in the final multivariable logistic regression models. NLR and procalcitonin remained independently associated with both mechanical ventilation requirement and 28-day mortality, whereas GCS score and hematoma volume were independently associated only with mechanical ventilation requirement. Confidence intervals crossing the null value (aOR = 1.0) indicate statistically non-significant associations. * *p* < 0.05.

The results of the multivariable logistic regression analyses are visually summarized in Figure 3. NLR and procalcitonin remained independently associated with both mechanical ventilation requirement and 28-day mortality, whereas GCS score and hematoma volume were independently associated only with mechanical ventilation requirement.

Because PIV and NLR were moderately to strongly correlated (ρ = 0.639, *p* < 0.001) and their simultaneous inclusion produced VIF values exceeding 6, only NLR was retained in the final multivariable models to reduce multicollinearity and model instability.

### 3.6. Correlation Analysis

Spearman correlation analysis demonstrated strong positive correlations between PIV and neutrophil count (ρ = 0.720, *p* < 0.001), NLR (ρ = 0.639, *p* < 0.001), and total leukocyte count (ρ = 0.690, *p* < 0.001). More modest correlations were observed with PLR, CRP, and procalcitonin. The complete correlation results are presented in Table 8.

## 4. Discussion

In this retrospective cohort of 111 critically ill patients with spontaneous ICH, we evaluated the prognostic value of PIV at ICU admission and compared it with established inflammatory indices and clinical scoring systems. The principal findings were: (i) PIV was significantly elevated in patients requiring MV but was not significantly associated with this outcome in univariable logistic regression and did not provide meaningful incremental value beyond the reference model; (ii) PIV showed poor discrimination for 28-day mortality (AUC = 0.535); (iii) NLR showed better discriminative performance than PIV and remained independently associated with both outcomes; and (iv) procalcitonin showed the highest observed discrimination among the evaluated inflammatory biomarkers for 28-day mortality and remained independently associated with both outcomes (aOR = 5.297).

The incremental model analyses further strengthen these findings. Addition of PIV did not meaningfully improve discrimination beyond the standard ICH score, NLR, and procalcitonin for either mechanical ventilation requirement or 28-day mortality. Although a nominal improvement in model fit was observed for mechanical ventilation, this was not accompanied by an increase in the c-statistic. Similarly, the small numerical increase in the c-statistic for 28-day mortality was not statistically significant. These findings emphasize that statistical association or improvement in model likelihood does not necessarily translate into clinically meaningful incremental prognostic value. Accordingly, the modest changes in model fit observed after inclusion of PIV should not be interpreted as evidence of clinically meaningful improvement in risk prediction.

The neuroinflammatory cascade following ICH involves rapid activation of resident microglia, extravasation of peripheral neutrophils into the perihematomal zone, monocyte-derived macrophage infiltration, and disruption of the blood–brain barrier, collectively promoting perihematomal edema and secondary neuronal injury [3,4,5]. Similar microglia-mediated processes, including blood–brain barrier disruption and cerebral edema, have also been described in other hemorrhagic stroke populations, such as subarachnoid hemorrhage [12]. This systemic immune perturbation is reflected in peripheral blood cell counts, forming the pathophysiological basis for inflammation-based prognostic indices. Our findings are consistent with previous evidence indicating that NLR, which captures the neutrophil–lymphocyte axis of this response, may provide prognostic information in critically ill patients with ICH [6,7,8]. In the present cohort, NLR remained independently associated with both study outcomes. Previous systematic reviews and meta-analyses have linked elevated admission NLR to 90-day mortality and unfavorable functional outcomes after spontaneous ICH [8]. However, heterogeneity in blood-sampling time, NLR thresholds, outcome definitions, and adjustment strategies limits comparisons across studies, while evidence beyond 90 days remains scarce [8]. Thus, although our 28-day findings are consistent with previous short- to medium-term observations, they do not establish the prognostic value of NLR for long-term functional recovery.

The limited incremental prognostic value of PIV over NLR in our cohort may be partly explained by the substantial statistical and mathematical overlap between these indices. PIV correlated strongly with NLR (ρ = 0.639, *p* < 0.001) and particularly with neutrophil count (ρ = 0.720, *p* < 0.001). These correlations are partly expected because neutrophil and lymphocyte counts are mathematical components of the PIV formula. Nevertheless, the findings suggest that the additional information contributed by monocytes and platelets did not translate into meaningful incremental prognostic value in this cohort. The inclusion of platelets in PIV may also introduce noise: platelet counts are influenced by multiple non-inflammatory factors in critically ill patients, including hemodilution, consumption coagulopathy, and medication effects. Monocyte counts may likewise be affected by concurrent infection, tissue injury, physiological stress, and the timing of blood sampling. Because PIV retains the same neutrophil–lymphocyte axis captured by NLR, the additional monocyte and platelet components can improve prognostic performance only if they provide outcome-relevant information that is not already represented by NLR. In the present cohort, these additional components did not translate into improved discrimination or meaningful incremental model performance. These observations are consistent with the heterogeneous literature on inflammatory indices in hemorrhagic stroke, in which simpler markers such as NLR have demonstrated prognostic value, while composite indices such as SIRI have also been associated with adverse outcomes in selected patient populations [13,14,15].

A notable finding was the independent association of admission procalcitonin with both MV requirement (aOR = 1.984) and 28-day mortality (aOR = 5.297, *p* < 0.001). Because pneumonia is an important complication after ICH [16], infection-related elevation of procalcitonin is clinically plausible in this population. Previous evidence concerning procalcitonin in primary ICH is not limited to acute in-hospital outcomes. In a prospective cohort of 251 patients with primary ICH, He et al. measured serum procalcitonin at admission and found that elevated levels were independently associated with both unfavorable functional outcome, assessed using the modified Rankin Scale, and mortality at 3 months [17]. These findings suggest that an early procalcitonin measurement may carry prognostic information beyond the immediate hospitalization. However, evidence extending beyond 3 months remains sparse, and the observed association should not be interpreted as an ICH-specific inflammatory mechanism. Procalcitonin is commonly used as a marker of bacterial infection [18]. However, elevated concentrations may also occur in non-infectious conditions characterized by severe sterile inflammation, tissue injury, surgery, shock, and organ dysfunction [19]. In our cohort—characterized by severe neurological injury and a high rate of ICH-related neurosurgical intervention (47.7%)—elevated procalcitonin at ICU admission may have reflected aspiration-related pneumonia or another concomitant systemic infection [16], organ dysfunction, or the generalized physiological stress response associated with critical illness rather than an ICH-specific neuroinflammatory signal. Because the temporal relationship between blood sampling and neurosurgical intervention could not be consistently determined, procedure-related inflammation could not be excluded in some patients. The absence of systematic microbiological surveillance data in our retrospective design precludes definitive attribution, and this finding warrants prospective investigation. From a clinical perspective, an elevated admission procalcitonin level may identify patients who warrant closer assessment for aspiration pneumonia, systemic infection, organ dysfunction, or severe physiological stress. However, a single procalcitonin measurement should not be interpreted as evidence of bacterial infection or used in isolation to guide antimicrobial treatment. Its interpretation should be integrated with clinical findings, microbiological data, imaging, organ-function parameters, and established measures of ICH severity.

Classical clinical variables—including GCS score, hematoma volume, and ICH score—showed strong associations with MV requirement, consistent with the established importance of neurological status and hematoma burden following ICH [1,2]. In the mortality model, these structural variables were no longer statistically significant after adjustment, whereas NLR and procalcitonin remained independently associated with mortality. This pattern suggests that, whereas the immediate need for ventilatory support was closely associated with acute neurological compromise, short-term mortality in this cohort was also associated with markers of systemic inflammation and physiological stress. However, these observational findings do not establish causality or demonstrate that modification of inflammatory biomarkers would improve clinical outcomes. Although GCS score, ICH score, and hematoma volume represent conceptually overlapping measures of neurological and radiological disease severity, formal collinearity diagnostics did not indicate problematic multicollinearity among the variables retained in the final models, with all VIF values below 3. Conversely, simultaneous inclusion of PIV and NLR produced VIF values exceeding 6, supporting exclusion of PIV from the final multivariable models. Nevertheless, the absence of high VIF values does not entirely eliminate the possibility of model instability in a relatively small cohort, and the multivariable findings should therefore be interpreted cautiously.

ICH-related neurosurgical intervention was significantly associated with both mechanical ventilation requirement and 28-day mortality in the univariable analyses. However, surgery was a treatment exposure occurring during the index hospitalization, and its temporal relationship with initiation of mechanical ventilation could not be consistently established. Moreover, the decision to perform surgery was likely influenced by hematoma characteristics and overall clinical severity, creating the possibility of confounding by indication. The very large odds ratio and wide confidence interval observed for mechanical ventilation also suggest sparse-data instability. Therefore, these univariable associations should not be interpreted as evidence of an independent prognostic or causal effect of surgery. Accordingly, surgery was not included in the final multivariable models.

Importantly, these findings should not be interpreted as indicating that inflammatory biomarkers supersede established clinical severity measures in prognostic assessment after ICH. The loss of statistical significance of GCS score, ICH score, and hematoma volume in the multivariable mortality model most likely reflects the limited sample size, the relatively low number of outcome events, and the correlation among markers of disease severity rather than a lack of prognostic importance. These established variables remain fundamental components of validated ICH prognostic models and should continue to form the basis of clinical risk stratification. Instead, our results suggest that inflammatory biomarkers, particularly NLR and procalcitonin, were independently associated with 28-day mortality in this cohort and may provide complementary prognostic information when interpreted alongside conventional clinical predictors.

From a clinical practice perspective, NLR and procalcitonin may be prioritized over PIV among inflammatory biomarkers considered for early risk stratification in ICU-admitted patients with ICH because they demonstrated better prognostic performance in this cohort. However, these biomarkers should complement rather than replace established neurological and radiological severity assessment. GCS and the ICH score should remain central to the initial prognostic evaluation, while NLR and procalcitonin may help identify patients who warrant closer monitoring and further assessment for systemic inflammation, infection, organ dysfunction, or physiological stress. NLR is universally available, inexpensive, and can be calculated without additional assays beyond the routine complete blood count. PIV, while conceptually appealing, does not appear to confer meaningful additional prognostic information in this critically ill population and involves an additional computational step requiring monocyte counts, which are not consistently available in all clinical settings.

Recent studies have increasingly focused on composite inflammatory biomarkers in hemorrhagic stroke [13,14,15]. Although these indices theoretically capture broader aspects of immune activation, our findings suggest that greater complexity did not translate into better prognostic performance in this cohort. Beyond blood count–derived indices, recent transcriptomic and single-cell studies have begun to characterize more complex peripheral immune signatures in ICH [5,20]. In hypertensive ICH, NXPE3 has recently been proposed as a candidate neutrophil-related biomarker for distinguishing hypertension from hypertensive ICH and for predicting ICH risk [20]. These emerging molecular approaches illustrate a shift toward higher-dimensional immune profiling; however, their clinical utility remains investigational and requires prospective validation. In critically ill patients with ICH, simpler markers such as NLR may therefore remain preferable because of their robustness, availability, and ease of clinical implementation.

## 5. Limitations

Several limitations of this study should be considered. First, the retrospective data collection and single-center design introduce the potential for selection and information bias and limit the generalizability of the findings to other ICU settings and patient populations. In addition, the cohort reflects the referral patterns, ICU admission criteria, intubation practices, surgical decision-making, and treatment protocols of a single institution. These institution-specific factors may have influenced both patient selection and the evaluated outcomes, thereby limiting the transportability of our findings to centers with different clinical practices and case mixes. Because ethnic diversity was not specifically evaluated, the applicability of the findings across ethnically and geographically diverse populations also remains uncertain. Second, inflammatory biomarkers were assessed at a single time point (ICU admission); dynamic changes in PIV, NLR, and procalcitonin during hospitalization—which may carry additional prognostic relevance—were not evaluated. Moreover, the exact interval from symptom onset to blood sampling and the timing of sampling relative to intubation and surgical or invasive procedures could not be consistently established from the retrospective records. Consequently, we could not distinguish immediate from delayed initiation of mechanical ventilation or evaluate whether the timing or duration of ventilation was associated with the studied outcomes. The temporal relationship between ICH-related neurosurgical intervention and initiation of mechanical ventilation could also not be consistently established; therefore, the observed associations involving surgery should not be interpreted as indicating a temporal or causal relationship. Third, despite bootstrap internal validation, the relatively small sample size and limited number of outcome events increase the possibility of overfitting and contribute to uncertainty in the estimated regression coefficients. With five predictors in each final model, the approximate events-per-predictor ratios were 15.6 for mechanical ventilation and 8.2 for 28-day mortality, indicating greater potential for coefficient instability in the mortality model. For the same reason, potential interactions between predictors were not evaluated, and the study was not powered to assess effect modification. Although collinearity diagnostics did not indicate problematic multicollinearity among the variables retained in the final models, the findings should be considered exploratory and require external validation in larger prospective cohorts. Bootstrap resampling provided internal assessment of model stability but cannot establish performance in independent populations. Consequently, the reported discrimination and regression estimates may be optimistic and should not be directly generalized to other institutions or used for clinical implementation before external validation. Fourth, standardized 90-day modified Rankin Scale scores and longer-term follow-up data were not systematically recorded. Therefore, although previous studies have associated admission NLR and procalcitonin with 3-month outcomes, the present study cannot determine whether these biomarkers predict medium- or long-term functional recovery or mortality beyond 28 days. Fifth, systematic microbiological data and standardized information regarding aspiration pneumonia, sepsis, and other concurrent infections were unavailable, precluding reliable distinction between infectious and non-infectious causes of procalcitonin elevation. Procalcitonin may also have been influenced by invasive procedures, surgical stress, organ dysfunction, or the generalized physiological response to critical illness. Furthermore, serial procalcitonin measurements were unavailable; therefore, we could not determine whether persistent or increasing concentrations were associated with evolving infection or whether isolated admission elevations represented a transient sterile inflammatory or physiological stress response.

## 6. Conclusions

In critically ill patients with spontaneous ICH, PIV measured at ICU admission demonstrated limited discriminative ability for MV requirement and showed poor discrimination for 28-day mortality. PIV did not provide meaningful incremental prognostic value beyond a reference model including the ICH score, NLR, and procalcitonin. Accordingly, our findings do not support the routine use of PIV for early risk stratification in ICU-admitted patients with spontaneous ICH. Its substantial overlap with NLR also limited the rationale for including both indices in the same final multivariable model. When inflammatory biomarkers are considered in this population, simpler markers—particularly NLR and procalcitonin—may be preferred over PIV because they demonstrated better prognostic performance. Given its simplicity, low cost, and universal availability, NLR may be the most practical among the inflammatory biomarkers evaluated in this study. Nevertheless, GCS and the ICH score should remain the foundation of clinical prognostic assessment, with NLR and procalcitonin interpreted as complementary markers rather than stand-alone determinants of management decisions. These findings should not be generalized beyond ICU-admitted patients with spontaneous ICH, and larger prospective multicenter studies with serial biomarker measurements, standardized functional outcomes, and external validation are required before broader clinical application.

## Figures and Tables

**Table 1:** Baseline characteristics of the study population according to mechanical ventilation requirement.

Variable	All Patients (*N* = 111)	No MV (*n* = 33)	MV Required (*n* = 78)	*p*
Age, years	69.0 (60.5–79.0)	65.0 (57.0–71.0)	72.0 (64.0–79.8)	0.028 *
Male sex, n (%)	68 (61.3)	23 (69.7)	45 (57.7)	0.330
GCS score	8 (4–13)	14 (13–15)	6 (3–9)	<0.001 *
Systolic BP, mmHg	138.0 (120.0–154.0)	150.0 (138.0–177.0)	130.0 (110.0–150.0)	<0.001 *
ICH score	2 (2–4)	2 (1–2)	3 (2–4)	<0.001 *
Hematoma volume, mL	84.0 (40.2–171.8)	44.0 (15.0–63.5)	114.0 (56.4–200.0)	<0.001 *
IVH, n (%)	55 (49.5)	12 (36.4)	43 (55.1)	0.110
Infratentorial location, n (%)	12 (10.8)	3 (9.1)	9 (11.5)	1.000
ICH-related surgery, n (%)	53 (47.7)	1 (3.0)	52 (66.7)	<0.001 *
WBC, ×10^9^/L	11.8 (8.9–15.0)	10.2 (8.5–13.5)	12.8 (9.3–15.2)	0.114
Neutrophil, ×10^9^/L	10.1 (7.2–13.1)	7.7 (6.5–11.0)	10.9 (8.2–13.4)	0.006 *
Lymphocyte, ×10^9^/L	1.0 (0.7–1.5)	1.5 (1.1–1.8)	0.8 (0.5–1.1)	<0.001 *
CRP, mg/L	29.0 (6.4–92.0)	8.5 (3.3–14.0)	47.0 (12.7–135.8)	<0.001 *
Procalcitonin, ng/mL	0.1 (0.0–0.7)	0.0 (0.0–0.1)	0.2 (0.1–1.2)	<0.001 *
PIV	901.6 (457.7–2019.9)	543.2 (434.9–1415.0)	1120.6 (562.4–2648.4)	0.013 *
NLR	11.3 (6.5–17.2)	5.8 (3.5–8.2)	13.3 (8.7–21.6)	<0.001 *
PLR	216.8 (159.9–323.3)	179.8 (128.2–240.3)	244.2 (169.8–404.1)	0.002 *

Continuous variables were compared using the Mann–Whitney U test. Categorical variables were compared using the Pearson chi-square test with Yates’ continuity correction, except for infratentorial location, for which Fisher’s exact test was used because of small expected cell counts. All tests were two-sided. ICH-related surgery was defined as a neurosurgical intervention performed for management of the index ICH during the index hospitalization. **Abbreviations:** GCS, Glasgow Coma Scale; ICH, intracerebral hemorrhage; IVH, intraventricular hemorrhage; WBC, white blood cell count; CRP, C-reactive protein; PIV, pan-immune-inflammation value; NLR, neutrophil-to-lymphocyte ratio; PLR, platelet-to-lymphocyte ratio; MV, mechanical ventilation. * *p* < 0.05.

**Table 2:** Baseline characteristics according to 28-day mortality.

Variable	All Patients (*N* = 111)	Survivors (*n* = 70)	Non-Survivors (*n* = 41)	*p*
Age, years	69.0 (60.5–79.0)	68.0 (57.3–76.5)	75.0 (67.0–80.0)	0.007 *
Male sex, n (%)	68 (61.3)	45 (64.3)	23 (56.1)	0.514
GCS score	8 (4–13)	12 (5–14)	6 (3–11)	0.018 *
Systolic BP, mmHg	138.0 (120.0–154.0)	142.5 (122.5–159.5)	130.0 (110.0–150.0)	0.010 *
ICH score	2 (2–4)	2 (1–3)	3 (2–4)	0.001 *
Hematoma volume, mL	84.0 (40.2–171.8)	56.0 (33.2–124.6)	130.0 (78.0–187.5)	<0.001 *
IVH, n (%)	55 (49.5)	31 (44.3)	24 (58.5)	0.210
Infratentorial location, n (%)	12 (10.8)	6 (8.6)	6 (14.6)	0.499
ICH-related surgery, n (%)	53 (47.7)	26 (37.1)	27 (65.9)	0.006 *
WBC, ×10^9^/L	11.8 (8.9–15.0)	11.6 (9.3–14.9)	12.2 (8.1–15.0)	0.741
Neutrophil, ×10^9^/L	10.1 (7.2–13.1)	9.9 (7.1–12.9)	10.2 (7.2–13.4)	0.783
Lymphocyte, ×10^9^/L	1.0 (0.7–1.5)	1.1 (0.8–1.7)	0.8 (0.4–1.1)	<0.001 *
CRP, mg/L	29.0 (6.4–92.0)	14.3 (3.8–84.2)	45.0 (12.2–108.0)	0.085
Procalcitonin, ng/mL	0.1 (0.0–0.7)	0.1 (0.0–0.2)	0.4 (0.1–1.6)	<0.001 *
PIV	901.6 (457.7–2019.9)	887.2 (455.4–1693.2)	1026.8 (554.8–2239.9)	0.539
NLR	11.3 (6.5–17.2)	8.2 (5.5–13.3)	13.8 (9.6–21.9)	0.001 *
PLR	216.8 (159.9–323.3)	206.4 (135.5–285.8)	249.1 (184.6–402.4)	0.039 *

Continuous variables were compared using the Mann–Whitney U test. Categorical variables were compared using the Pearson chi-square test with Yates’ continuity correction, except for infratentorial location, for which Fisher’s exact test was used because of small expected cell counts. All tests were two-sided. ICH-related surgery was defined as a neurosurgical intervention performed for management of the index ICH during the index hospitalization. **Abbreviations:** GCS, Glasgow Coma Scale; ICH, intracerebral hemorrhage; IVH, intraventricular hemorrhage; WBC, white blood cell count; CRP, C-reactive protein; PIV, pan-immune-inflammation value; NLR, neutrophil-to-lymphocyte ratio; PLR, platelet-to-lymphocyte ratio. * *p* < 0.05.

**Table 3:** ROC performance of clinical and inflammatory predictors for mechanical ventilation requirement.

Marker	AUC (95% CI)	Cutoff	Sensitivity (%)	Specificity (%)	Youden
GCS	0.922 (0.858–0.974)	≤10.00	78.2	97.0	0.752
ICH score	0.874 (0.773–0.908)	≥3.00	66.7	93.9	0.606
Hematoma volume	0.797 (0.712–0.877)	≥76.50	69.2	78.8	0.480
NLR	0.832 (0.749–0.903)	≥11.69	64.1	93.9	0.580
PLR	0.689 (0.587–0.781)	≥288.41	39.7	93.9	0.337
PIV	0.650 (0.544–0.753)	≥665.72	73.1	63.6	0.367
Procalcitonin	0.861 (0.774–0.934)	≥0.06	88.5	75.8	0.642

AUC confidence intervals were estimated using percentile bootstrap resampling with 2000 iterations. Optimal cutoff values were determined using the Youden index. **Abbreviations:** AUC, area under the curve; CI, confidence interval; GCS, Glasgow Coma Scale; ICH, intracerebral hemorrhage; PIV, pan-immune-inflammation value; NLR, neutrophil-to-lymphocyte ratio; PLR, platelet-to-lymphocyte ratio.

**Table 4:** ROC performance of clinical and inflammatory predictors for 28-day mortality.

Marker	AUC (95% CI)	Cutoff	Sensitivity (%)	Specificity (%)	Youden
GCS	0.633 (0.535–0.738)	≤11.00	80.5	50.0	0.305
ICH score	0.672 (0.579–0.777)	≥3.00	70.7	64.3	0.350
Hematoma volume	0.691 (0.589–0.784)	≥78.00	75.6	58.6	0.342
NLR	0.687 (0.586–0.790)	≥12.91	63.4	72.9	0.363
PLR	0.618 (0.502–0.730)	≥195.33	73.2	48.6	0.217
PIV	0.535 (0.418–0.647)	≥722.40	68.3	47.1	0.154
Procalcitonin	0.800 (0.723–0.874)	≥0.12	85.4	64.3	0.497

AUC confidence intervals were estimated using percentile bootstrap resampling with 2000 iterations. Optimal cutoff values were determined using the Youden index. **Abbreviations:** AUC, area under the curve; CI, confidence interval; GCS, Glasgow Coma Scale; ICH, intracerebral hemorrhage; PIV, pan-immune-inflammation value; NLR, neutrophil-to-lymphocyte ratio; PLR, platelet-to-lymphocyte ratio.

**Table 5:** Univariable logistic regression for mechanical ventilation requirement.

Variable	Unit	OR (95% CI)	*p*
Age	per 1-SD	1.47 (0.98–2.22)	0.065
GCS	per 1-SD	0.06 (0.02–0.19)	<0.001 *
Hematoma volume	per 1-SD	7.71 (2.71–21.96)	<0.001 *
ICH score	per 1-SD	5.94 (2.86–12.36)	<0.001 *
IVH	yes/no	2.15 (0.93–4.97)	0.073
ICH-related surgery	yes/no	64.00 (8.28–494.82)	<0.001 *
PIV	per 1-SD log	1.49 (0.96–2.32)	0.074
NLR	per 1-SD log	4.64 (2.42–8.89)	<0.001 *
PLR	per 1-SD log	1.96 (1.19–3.24)	0.009 *
Procalcitonin	per 1-SD log	19.43 (2.55–147.92)	0.004 *
Lymphocyte	per 1-SD	0.47 (0.24–0.93)	0.030 *
Neutrophil	per 1-SD	2.04 (1.16–3.57)	0.013 *
CRP	per 1-SD log	2.56 (1.57–4.20)	<0.001 *

Odds ratios and two-sided *p*-values were obtained from univariable binary logistic regression models using Wald tests. ORs for continuous predictors are reported per one-standard-deviation increase. For variables designated “per 1-SD log,” values were first transformed using ln(x + 1) and subsequently standardized. Binary predictors are reported for presence versus absence. ICH-related surgery was defined as a neurosurgical intervention performed for management of the index ICH during the index hospitalization. **Abbreviations:** OR, odds ratio; CI, confidence interval; GCS, Glasgow Coma Scale; ICH, intracerebral hemorrhage; PIV, pan-immune-inflammation value; NLR, neutrophil-to-lymphocyte ratio; PLR, platelet-to-lymphocyte ratio. * *p* < 0.05.

**Table 6:** Univariable logistic regression for 28-day mortality.

Variable	Unit	OR (95% CI)	*p*
Age	per 1-SD	1.77 (1.14–2.77)	0.012 *
GCS	per 1-SD	0.60 (0.40–0.91)	0.015 *
Hematoma volume	per 1-SD	1.83 (1.18–2.82)	0.006 *
ICH score	per 1-SD	1.95 (1.26–3.01)	0.003 *
IVH	yes/no	1.78 (0.81–3.87)	0.149
ICH-related surgery	yes/no	3.26 (1.46–7.32)	0.004 *
PIV	per 1-SD log	1.05 (0.71–1.55)	0.807
NLR	per 1-SD log	2.09 (1.29–3.38)	0.003 *
PLR	per 1-SD log	1.61 (1.05–2.47)	0.028 *
Procalcitonin	per 1-SD log	3.39 (1.73–6.65)	<0.001 *
Lymphocyte	per 1-SD	0.26 (0.11–0.63)	0.003 *
Neutrophil	per 1-SD	1.10 (0.75–1.62)	0.613
CRP	per 1-SD log	1.44 (0.97–2.15)	0.072

Odds ratios and two-sided *p*-values were obtained from univariable binary logistic regression models using Wald tests. ORs for continuous predictors are reported per one-standard-deviation increase. For variables designated “per 1-SD log,” values were first transformed using ln(x + 1) and subsequently standardized. Binary predictors are reported for presence versus absence. ICH-related surgery was defined as a neurosurgical intervention performed for management of the index ICH during the index hospitalization. **Abbreviations:** OR, odds ratio; CI, confidence interval; GCS, Glasgow Coma Scale; ICH, intracerebral hemorrhage; PIV, pan-immune-inflammation value; NLR, neutrophil-to-lymphocyte ratio; PLR, platelet-to-lymphocyte ratio. * *p* < 0.05.

**Table 7:** Multivariable logistic regression models for mechanical ventilation requirement and 28-day mortality.

Variable	aOR (95% CI)	*p*	aOR (95% CI)	*p*
	**Mechanical Ventilation Requirement**		**28-Day Mortality**	
GCS score	0.200 (0.087–0.415)	<0.001 *	1.202 (0.661–2.054)	0.525
ICH score	1.733 (0.917–3.570)	0.113	1.771 (0.953–3.340)	0.074
Hematoma volume	2.200 (1.202–4.443)	0.018 *	1.270 (0.788–2.010)	0.317
NLR	2.430 (1.288–4.870)	0.009 *	3.192 (1.271–7.652)	0.011 *
Procalcitonin	1.984 (1.298–3.132)	0.002 *	5.297 (2.184–10.100)	<0.001 *

Adjusted odds ratios and two-sided *p*-values were obtained from multivariable binary logistic regression models using Wald tests. Confidence intervals were estimated using bootstrap resampling with 2000 iterations. **Model performance:** Mechanical ventilation model, c-statistic = 0.944; 28-day mortality model, c-statistic = 0.809. Adjusted odds ratios for GCS score, ICH score, and hematoma volume represent a one-standard-deviation increase in the original variable. NLR and procalcitonin were transformed using ln(x + 1) and standardized; their adjusted odds ratios therefore represent a one-standard-deviation increase in the transformed value. **Abbreviations:** aOR, adjusted odds ratio; CI, confidence interval; GCS, Glasgow Coma Scale; ICH, intracerebral hemorrhage; NLR, neutrophil-to-lymphocyte ratio. Models were adjusted for GCS score, ICH score, hematoma volume, NLR, and procalcitonin. Collinearity diagnostics were acceptable for all retained predictors, with VIF values ranging from 1.01 to 2.93. * *p* < 0.05.

**Table 8:** Spearman correlation of PIV with inflammatory markers.

Variable	Spearman ρ with PIV	*p*
NLR	0.639	<0.001 *
PLR	0.458	<0.001 *
Neutrophil	0.720	<0.001 *
Lymphocyte	−0.201	0.034 *
WBC	0.690	<0.001 *
Platelet	0.353	<0.001 *
Procalcitonin	0.229	0.016 *
CRP	0.254	0.007 *

**Abbreviations:** PIV, pan-immune-inflammation value; NLR, neutrophil-to-lymphocyte ratio; PLR, platelet-to-lymphocyte ratio; CRP, C-reactive protein; WBC, white blood cell count. * *p* < 0.05.

## Data Availability

The data supporting the findings of this study are available from the corresponding author upon reasonable request, subject to institutional data-sharing regulations.

## Supplementary Materials

The following supporting information can be downloaded at: https://www.mdpi.com/article/10.3390/brainsci16080842/s1, Table S1. Pairwise bootstrap-based comparisons of ROC curves between PIV and selected prognostic markers. Table S2. Incremental prognostic performance of reference models with and without PIV. Table S3. Collinearity diagnostics for predictors considered in the multivariable logistic regression models.
