# Supplementary material for: Limited Prognostic Value of Pan-Immune-Inflammation Value Compared with NLR and Procalcitonin in Critically Ill Patients with Spontaneous Intracerebral Hemorrhage: A Retrospective Cohort Study
Source: Brain Sci. 2026 Aug 8;16(8):842. doi: 10.3390/brainsci16080842 (PMC13511428; doi:10.3390/brainsci16080842)
Supplement: Supplementary file 1 [file brainsci-16-00842-s001.zip › Table S1.pdf]

Supplementary Table S1. Pairwise bootstrap-based comparisons of ROC curves between PIV and selected prognostic markers.

Outcome	Comparison	AUC Difference	p
MV requirement	NLR vs PIV	0.183	0.0002*
MV requirement	GCS vs PIV	0.266	<0.0001*
MV requirement	ICH score vs PIV	0.220	0.0006*
28-day mortality	NLR vs PIV	0.152	0.0016*
28-day mortality	GCS vs PIV	0.103	0.167
28-day mortality	ICH Score vs PIV	0.141	0.082

Pairwise AUC comparisons were performed using two-sided bootstrap-based testing with 2,000 resamples. **Abbreviations:** AUC, area under the curve; PIV, pan-immune-inflammation value; NLR, neutrophil-to-lymphocyte ratio; GCS, Glasgow Coma Scale; ICH, intracerebral hemorrhage. * $p < 0.05$.
