# Supplementary material for: Limited Prognostic Value of Pan-Immune-Inflammation Value Compared with NLR and Procalcitonin in Critically Ill Patients with Spontaneous Intracerebral Hemorrhage: A Retrospective Cohort Study
Source: Brain Sci. 2026 Aug 8;16(8):842. doi: 10.3390/brainsci16080842 (PMC13511428; doi:10.3390/brainsci16080842)
Supplement: Supplementary file 1 [file brainsci-16-00842-s001.zip › Table S2.pdf]

Supplementary Table S2. Incremental prognostic performance of reference models with and without PIV.

Outcome	Model	AUC	ΔAUC	Bootstrap AUC comparison p	Likelihod-ratio test p	Brier score	Hosmer–Lemeshow p
Mechanical ventilation	Reference model	0.916	—	—	—	0.107	0.168
Mechanical ventilation	Reference model + PIV	0.916	−0.0004	0.463	0.044*	0.106	0.808
28-day mortality	Reference model	0.789	—	—	—	0.173	0.654
28-day mortality	Reference model + PIV	0.811	+0.023	0.268	0.052	0.167	0.529

Nested reference and extended logistic regression models were compared using the likelihood-ratio chi-square test. Differences in AUC between the reference and extended models were assessed using two-sided bootstrap-based testing with 2,000 resamples. The Brier score was calculated as the mean squared difference between observed outcomes and predicted probabilities. Model calibration was assessed using the Hosmer–Lemeshow goodness-of-fit test. All reported hypothesis tests were two-sided. The reference model included the standard ICH score, log-transformed NLR, and log-transformed procalcitonin. The extended model additionally included log-transformed PIV. ΔAUC represents the difference between the extended and reference models. Abbreviations: AUC, area under the curve; PIV, pan-immune-inflammation value; LR, likelihood-ratio; NLR, neutrophil-to-lymphocyte ratio; ICH, intracerebral hemorrhage. * $p < 0.05$.
