# Supplementary material for: Limited Prognostic Value of Pan-Immune-Inflammation Value Compared with NLR and Procalcitonin in Critically Ill Patients with Spontaneous Intracerebral Hemorrhage: A Retrospective Cohort Study
Source: Brain Sci. 2026 Aug 8;16(8):842. doi: 10.3390/brainsci16080842 (PMC13511428; doi:10.3390/brainsci16080842)
Supplement: Supplementary file 1 [file brainsci-16-00842-s001.zip › Table S3.pdf]

Supplementary Table S3. Collinearity diagnostics for predictors considered in the multivariable logistic regression models.

Predictor	Final model VIF	Tolerance	VIF when PIV and NLR were entered together
GCS score	2.73	0.366	2.75
ICH score	2.93	0.341	2.94
Hematoma volume	1.28	0.782	1.35
NLR	1.01	0.986	6.29
Procalcitonin	1.06	0.948	1.07
PIV	Not included	Not applicable	6.28

Multicollinearity was assessed using variance inflation factors (VIFs) and tolerance values, with tolerance calculated as 1/VIF. Diagnostics were calculated for the predictors retained in the final multivariable models and for an additional predictor set in which PIV and NLR were entered simultaneously. A VIF < 5 and a tolerance value > 0.20 were considered indicative of acceptable collinearity. VIF and tolerance are diagnostic measures rather than inferential hypothesis tests; therefore, no p-values were calculated. VIF values were calculated using the study dataset of 111 patients. The final multivariable models included GCS score, ICH score, hematoma volume, NLR, and procalcitonin. PIV was not included in the final models because its simultaneous inclusion with NLR increased the VIF values of both inflammatory indices above 6. Abbreviations: VIF, variance inflation factor; GCS, Glasgow Coma Scale; ICH, intracerebral hemorrhage; NLR, neutrophil-to-lymphocyte ratio; PIV, pan-immune-inflammation value.
